# Dislodged Right Ventricular Pacemaker Lead Detected Incidentally Two Years Post-implantation via Echocardiography: A Case Report

**DOI:** 10.7759/cureus.89305

**Published:** 2025-08-03

**Authors:** Buthainah Alhwarat, Khalid Sawalha, Ibrahim Alshaghdali, Abdelmoniem Moustafa, Hakan Paydak

**Affiliations:** 1 Internal Medicine, University of Arkansas for Medical Sciences, Little Rock, USA; 2 Cardiovascular Disease, University of Arkansas for Medical Sciences, Little Rock, USA; 3 Cardiometabolic Medicine, University of Missouri Kansas City School of Medicine, Kansas City, USA; 4 Clinical Cardiac Electrophysiology, University of Arkansas for Medical Sciences, Little Rock, USA

**Keywords:** dual-chamber pacemaker, echo cardiogram, incidental, lead misplacement, right ventricular outflow tract

## Abstract

Dislodgement of cardiac implantable electronic device (CIED) leads following implantation ideally should not be missed. However, more often than not, patients do not undergo post-operative imaging following these procedures due to a lack of evidence behind the role of imaging following uncomplicated pacemaker (PPM) implantation procedures, based on the most recent guidelines*.* This is a case report of a dual-chamber pacemaker (DC-PPM) right ventricular (RV) lead that was found dislodged and coiled in the right ventricular outflow tract (RVOT) as an incidental finding via a routine transthoracic echocardiogram (TTE) study two years after implantation. We intend to shed light on the significance of timely detection of lead-related complications, as they can result in poor outcomes for patients.

## Introduction

Cardiac pacemakers, a type of cardiac implantable electronic device (CIED), function as devices that are often implanted as a treatment for cardiac conduction abnormalities [1]. Pacemaker leads are connected to the myocardium in one of two ways: actively fixed through a helix on the distal end of a given lead, or passively, where leads are anchored to the myocardium via tines [1]. Pacemakers have evolved to operate with the function of sensing, where the device lead is able to sense intrinsic activity within the chamber that it is located in, in addition to their original function of pacing the myocardium [1]. Single-chamber pacing is most commonly achieved through placing a lead in the right ventricle (RV), placing the tip of the lead in the apex of the RV, whereas dual-chamber pacing is achieved through placing an additional lead in the right atrium, specifically the right atrial appendage [2].

As mentioned previously, CIED implantation procedures include the advancement of either one or two leads into cardiac chambers (the right atrium and ventricle in the case of our patient) via a cephalic, axillary, or subclavian vein [3]. However, multiple cases have been previously reported of erroneous dislodgement of pacer leads, either within the same original cardiac chamber or to a different one, with multiple cases documenting leads dislodged into the left ventricle (LV) chamber today [4]. Studies have shown that in conventional pacemaker implants, lead dislodgement occurs in 1-2% of cases (weighted mean ~1.7%) [5]. Common patterns of lead dislodgement include lead migration into the right atrium, superior vena cava, or pulmonary artery. Mechanical causes such as "twiddler’s syndrome," where patient manipulation leads to lead retraction, or "reel syndrome," involving rotation of the pulse generator, can also contribute. Recognition of these patterns is critical for timely diagnosis and intervention [6, 7].

In the case we report, the indication for a pacemaker placement was a complete and irreversible atrioventricular block. In these cases, dual-chamber pacing (the right atrium and ventricle) is the gold standard treatment in patients with preserved atrial contraction. For our 49-year-old patient with complete atrioventricular block, the RV lead was aimed to be placed at the right ventricular septum during the implantation procedure (October 2017). It was not until August 2019 - two years later - that the RV lead was found to be in an abnormally coiled position in the right ventricular outflow tract (RVOT) during a follow-up echocardiography (TTE) exam that was performed on an outpatient basis. Lead misplacement can result in serious complications, including tricuspid regurgitation due to valvular interference and ineffective pacing secondary to suboptimal myocardial engagement [8, 9]. Only a few cases have been reported of missed CIED lead dislodgement that was found years after implantation - a rare but potentially life-threatening complication of pacemaker device placement, which was, in our case, discovered during routine cardiac echocardiography.

## Case presentation

The patient we present is a 49-year-old female with a past medical history significant for primary hypertension, non-ischemic cardiomyopathy, and recurrent laryngeal papillomatosis. Moreover, she had a history of complete heart block for which no identifiable reversible cause was found. Therefore, a dual-chamber pacemaker (DC-PPM) was implanted in 2017.

At the time, the patient was scheduled to receive regular device interrogations through the device clinic. The first device interrogation following her procedure was in 12/2018 and revealed a predominant atrial paced-ventricular paced mode (73% of the time over a period of 3 months) with a 99% RV pacing percentage. RV lead impedance ranged between 500 and 700 ohms, correlating with a normal impedance range. Moreover, she had one documented episode of non-sustained ventricular tachycardia, with no other acute events documented. The next device interrogation was in 05/2019 and revealed largely similar parameters, including RV lead impedance, and two acute events consisting of one atrial fibrillation episode lasting 2 minutes and three atrial tachycardia episodes lasting 5 minutes at the longest. This finding is significant because it shows device data may not always reveal CIED lead-related complications.

In 2019, the patient was admitted for a left heart catheterization upon presentation with typical anginal chest pain. A subsequent single-photon emission computed tomography (SPECT) scan showed a moderate-sized, moderate-intensity fixed perfusion defect along the anterior wall of the left ventricle and a low estimated left ventricular ejection fraction (LVEF) of 20% as a result. Subsequently, the patient underwent a left-heart catheterization procedure, which only showed mild coronary artery disease (CAD), and there were no comments regarding the location or the position of the DC-PPM leads in the procedure note. The patient was thereafter discharged, and a plan to obtain an echocardiography to evaluate for new-onset heart failure/cardiomyopathy as an outpatient procedure was made.

**Figure 1:**
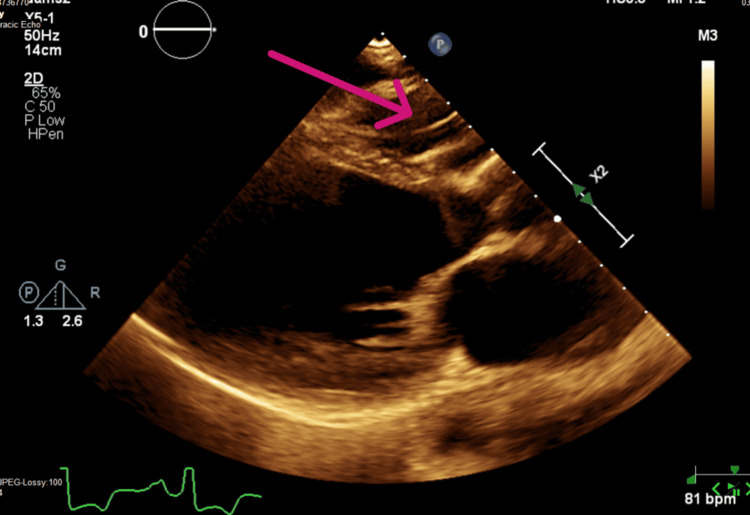
Para-sternal long axis view of TTE study performed in 08/2019 showing right ventricle (RV) pacemaker lead in the right ventricular outflow tract (RVOT) (arrow). Two-dimensional transthoracic echocardiography (TTE) studies were performed using commercially available systems (Vivid 7 and 9, GE Medical Systems, Milwaukee, WI, USA). Images were obtained with the patient in the left lateral decubitus position and in the supine position using a 3.5-MHz transducer. Standard two-dimensional, color, pulsed, and continuous-wave Doppler data were acquired digitally during gently held end-expiration and saved in regular cine loop format for subsequent offline analysis (EchoPAC version 111.0.00; GE-Vingmed Ultrasound AS, Asker, Norway).

**Figure 2:**
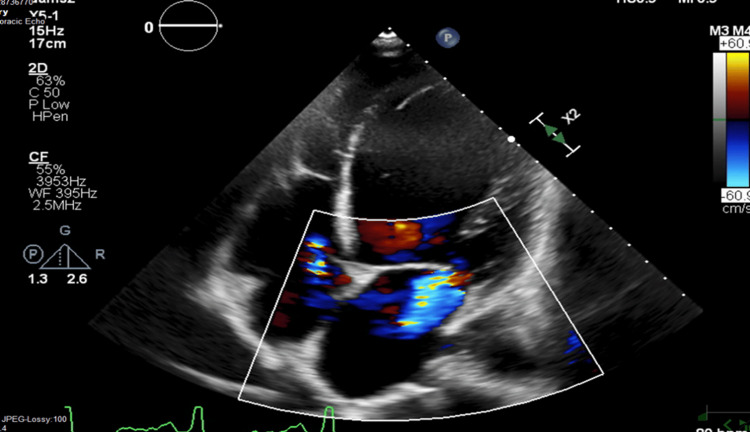
Four chamber view of TTE study performed in 08/2019 showing evidence of tricuspid valve regurgitation as a result of pacemaker right ventricle (RV) lead placement. Two-dimensional transthoracic echocardiography (TTE) studies were performed using commercially available systems (Vivid 7 and 9, GE Medical Systems, Milwaukee, WI, USA). Images were obtained with the patient in the left lateral decubitus position and in the supine position using a 3.5-MHz transducer. Standard two-dimensional, color, pulsed, and continuous-wave Doppler data were acquired digitally during gently held end-expiration and saved in regular cine loop format for subsequent offline analysis (EchoPAC version 111.0.00; GE-Vingmed Ultrasound AS, Asker, Norway).

**Figure 3:**
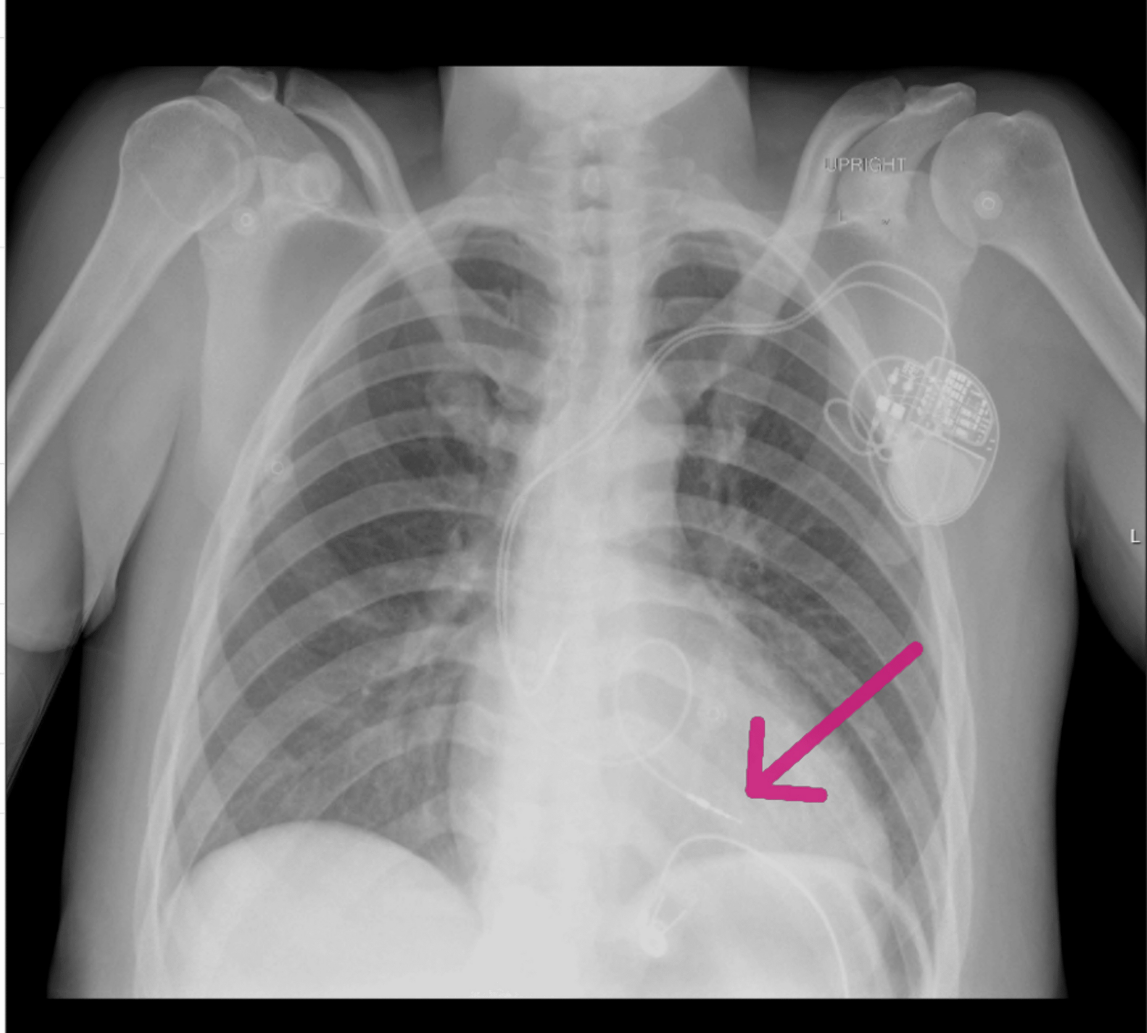
Chest X-ray study from 03/2019 showing a grossly coiled, misplaced right ventricle (RV) pacer lead in right ventricular outflow tract (RVOT) (arrow).

A TTE soon after discharge confirmed heart failure with reduced left ventricular ejection fraction (HFrEF) with an LVEF of 25% to 30%; however, this was not the only significant finding this examination helped identify. After the performing cardiologist was initially unable to locate the RV pacer lead anywhere within the RV chamber - its original implantation site - from either the four-chamber or apical views, he was able to identify it coiled in the RVOT - an unforeseen finding two years following implantation (Figures 1, 2). A chest radiograph was then obtained, which confirmed the TTE finding of a coiled and abnormally located RV lead in the RVOT (Figure 3).

**Figure 4:**
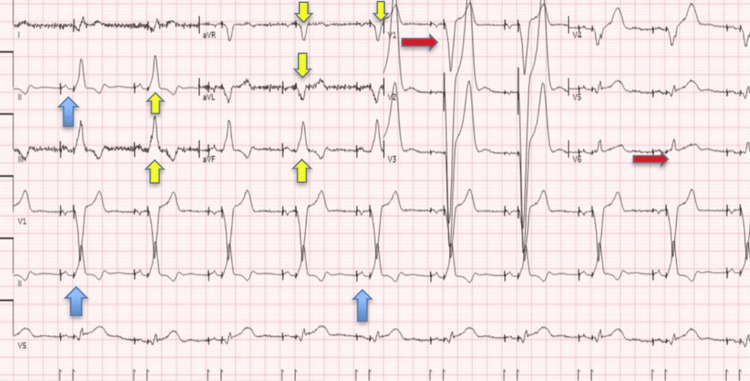
EKG study from 10/2017. EKG study from 10/2017 showing ventricular pacing (blue arrows) and evidence of left bundle branch block (red arrows) and associated right axis deviation indicating pacing of the right ventricular outflow tract (RVOT) at the time of this EKG with negative paced QRS complexes in leads V1, aVR, and aVL and positive QRS position in inferior leads (yellow arrows).

**Figure 5:**
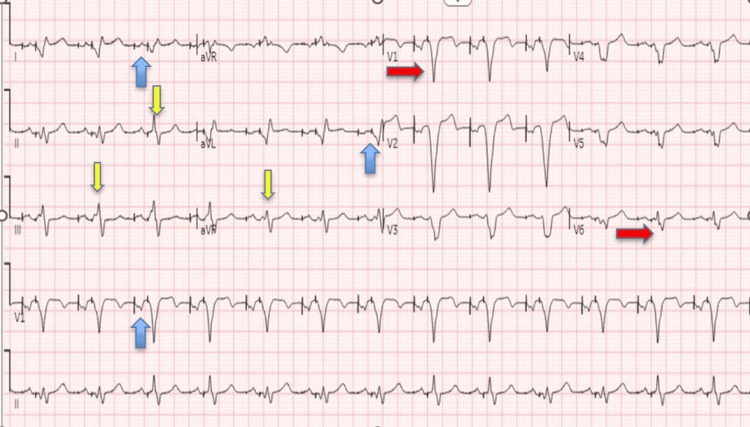
EKG study from 09/2020. EKG study from 09/2020 showing atrioventricular-dual-paced rhythm with evidence of atrial and ventricular (in some leads) pacing (blue arrows) and left bundle branch block (red arrows) and right axis deviation, as the inferior leads now show a positive/negative component (yellow arrows).

An electrocardiogram (EKG) study from September 2020 also showed atrioventricular-dual-paced rhythm with evidence of RVOT pacing in the ventricle (Figure 4), a finding that was missed on an EKG performed immediately after the procedure (October 2017) (Figure 5).

Based on our patient’s elevated percentage of ventricular pacing on device interrogation and after an ischemic workup with a left heart catheterization to rule out ischemic cardiomyopathy and a workup for myocarditis that was negative, it was decided that an upgrade procedure would be of higher benefit than lead repositioning to minimize her risk of adverse procedural outcomes. However, in light of the pulmonary condition due to increased risk of infection and complex anatomy that would increase her risk of operative complications, the upgrade procedure was postponed till January 2022, when a failed attempt was made to insert an LV lead due to difficulty engaging the coronary sinus (CS) to insert the LV lead.

Another unsuccessful attempt was made in January 2023 to place a left bundle branch area and/or coronary sinus lead. After cardiac CT showed a prominent Thebesian valve, likely explaining the difficulty gaining access to CS, a plan was made to refer the patient to cardiothoracic surgery for epicardial LV lead placement through an open surgery approach.

At this time, our patient continues to have poor candidacy for surgery given her underlying respiratory condition, increasing her risk of complications from a major procedure requiring general anesthesia, and she will need clearance from the otolaryngology and oncology teams prior to the anticipated upgrade to biventricular PPM surgery.

## Discussion

Lead dislodgement is not an uncommon complication following cardiac CIED implantation procedures, with a burden of long-term lead dislodgement ranging between 1.8% and 8% [8, 9]. These events are associated with an increased risk of serious adverse outcomes or complications in patients with CIEDs [10]. In a retrospective cohort analysis of 20,683 patients who underwent CIED implantation, Qin et al. identified female sex and a high body mass index as two patient-related risk factors for an increased risk of lead dislodgement of CIEDs [10].

With ongoing evolutions in the world of cardiac electrophysiology and advancements of CIED procedures, particularly the wide use of active fixation electrodes, it has become increasingly common to place pacer leads in the RVOT, granting a pacing location closer to the atrioventricular (AV) node and thus facilitating ‘a more natural’ pattern of contraction [11-14]. However, in our case report, the RV pacer lead was incidentally found to be coiled in the RVOT during an outpatient follow-up TTE study, two years after the original implantation procedure. Of note, it was planned for the RV lead to be placed near the RV apex for this case we report. This resulted in EKG abnormalities as described earlier. Moreover, a follow-up chest X-ray confirmed this lead dislodgement, confirming the value of this simple yet informative postoperative imaging modality. 

Routine imaging is not included among standard recommendations for CIED placement based on the 2024 American Heart Association/American College of Cardiology (AHA and ACC) multi-society guideline on perioperative cardiovascular management for noncardiac surgery [15]. Instead, chest radiography should be used selectively, based on clinical need. As evident in our case, some CIED lead-related complications may go unnoticed for years; therefore, it may be indicated to reconsider post-procedural imaging practices for these patients.

Similar cases of CIED-lead dislodgements have been previously documented through case reports with complications ranging from incidental findings of unrecognized complications by routine cardiac imaging, like echocardiography, to serious complications like systemic thromboembolism.

Many studies have documented the diagnostic value of transthoracic echocardiogram studies (TTE) in detecting CIED lead malposition or dislodgement. In fact, it has been named the imaging modality of choice to identify the exact position of CIED leads and delineate their routes [16-19]. This underscores the necessity of remaining vigilant for CIED-related complications in these patient groups during echocardiogram studies and electrocardiograms. Early identification and intervention are crucial in preventing these adverse outcomes.

## Conclusions

CIED lead-related complications are not uncommon and range from incidental findings on cardiac imaging modalities like echocardiography or studies like electrocardiograms to life-threatening dislodgement and systemic thromboembolism of these leads, which raises concern for a higher-than-estimated burden of undiagnosed CIED lead-related complications. In our patient's case, she was found to have RV lead dislodgement in an incidental fashion during a routine echocardiogram two years following device implantation. With less evidence for the role of routine post-implantation chest X-rays, this could possibly entail an increased burden of adverse outcomes for patients with CIEDs. This raises the question of whether guidelines should reconsider post-CIED implantation CXR, a widely accessible study, to evaluate for immediate post-procedural complications, including CIED lead-related ones. Lastly, the use of cardiac imaging modalities, particularly echocardiograms, can help identify lead malposition early and facilitate timely re-fixation and/or treatment with anticoagulation, if indicated.
